# Intergenerational Effects of Discrimination on Black American Children’s Sleep Health

**DOI:** 10.3390/ijerph19074021

**Published:** 2022-03-28

**Authors:** Madeleine F. Cohen, Anne L. Dunlop, Dayna A. Johnson, Alexis Dunn Amore, Elizabeth J. Corwin, Patricia A. Brennan

**Affiliations:** 1 Department of Psychology, Emory University, Atlanta, GA 30322, USA; pbren01@emory.edu; 2 Department of Gynecology and Obstetrics, School of Medicine, Emory University, Atlanta, GA 30322, USA; amlang@emory.edu; 3 Department of Epidemiology, Emory University, Atlanta, GA 30322, USA; dayna.johnson@emory.edu; 4 Nell Hodgson Woodruff School of Nursing, Emory University, Atlanta, GA 30322, USA; alexis.dunn.amore@emory.edu; 5 School of Nursing, Columbia University, New York, NY 10032, USA; ejc2202@cumc.columbia.edu

**Keywords:** discrimination, prenatal depressive symptoms, prenatal sleep quality, early childhood sleep health

## Abstract

Greater exposure to racial/ethnic discrimination among pregnant Black American women is associated with elevated prenatal depressive symptomatology, poorer prenatal sleep quality, and poorer child health outcomes. Given the transdiagnostic importance of early childhood sleep health, we examined associations between pregnant women’s lifetime exposure to racial/ethnic discrimination and their two-year-old children’s sleep health. We also examined women’s gendered racial stress as a predictor variable. In exploratory analyses, we examined prenatal sleep quality and prenatal depressive symptoms as potential mediators of the prior associations. We utilized data from a sample of Black American women and children (*n* = 205). Women self-reported their lifetime experiences of discrimination during early pregnancy, their sleep quality and depressive symptoms during mid-pregnancy, and their children’s sleep health at age two. Hierarchical linear multiple regression models were fit to examine direct associations between women’s experiences of discrimination and children’s sleep health. We tested our mediation hypotheses using a parallel mediator model. Higher levels of gendered racial stress, but not racial/ethnic discrimination, were directly associated with poorer sleep health in children. Higher levels of racial/ethnic discrimination were indirectly associated with poorer sleep health in children, via women’s prenatal depressive symptomatology, but not prenatal sleep quality. Clinical efforts to mitigate the effects of discrimination on Black American women may benefit women’s prenatal mental health and their children’s sleep health.

## 1. The Current Study

Greater exposure to racial/ethnic discrimination is associated with poorer health outcomes in Black American children and adults [1]. As a function of their overlapping and interrelated racial/ethnic and gender identities, Black American women are disproportionately exposed to and affected by multiple interpersonal psychosocial stressors, including racial/ethnic discrimination and sexism [2,3]. Proponents of intersectionality theory emphasize that Black American women often experience racial/ethnic discrimination and sexism simultaneously [2,3,4,5]. This co-occurrence of stressors has been termed gendered racism [6] and researchers have argued that studies examining associations between Black American women’s exposure to racial/ethnic discrimination and health outcomes should also include women’s exposure to gendered racism as a predictor variable, when possible [4]. Black American women who report higher levels of lifetime exposure to racial/ethnic discrimination report poorer mental and physical health outcomes [7]. Higher levels of gendered racism are also associated with poorer mental and physical health in Black American women [4], although fewer studies have examined these associations. Importantly, these stress exposures affect not only the health of Black American women themselves, but also hold implications for child health outcomes.

In recent years, more attention has been paid to the Barker Hypothesis [8], a conceptual framework that posits that higher levels of psychological distress experienced prior to and during a women’s pregnancy may place her child at risk for a range of negative developmental sequelae [9]. It is important to apply this conceptual framework to design and conduct studies of minority health that span across a woman’s pregnancy and into her offspring’s childhood. Black American women in particular are vulnerable to significant contextual stressors, which may place their children at greater risk for negative health outcomes. Indeed, greater lifetime exposure to racial/ethnic discrimination among pregnant Black American women is associated with an increased likelihood of preterm birth or low birthweight [10,11]. The range of childhood health outcomes that have been studied within this intergenerational framework has largely been confined to markers of child health within the neonatal period [10,11,12]. However, it is unlikely that these effects are restricted to early development. Further research is needed to understand how greater lifetime exposure to racial/ethnic discrimination or gendered racial stress among pregnant Black American women is adversely associated with aspects of children’s health further in development. The current study aims to address this gap by focusing on a transdiagnostic marker of pediatric health, early childhood sleep health.

Sleep health during early childhood is an important predictor of children’s temperament, cognition, behavior, and cardiometabolic functioning [13]. To our knowledge, only one study to date has investigated expectant mothers’ lifetime exposure to racial/ethnic discrimination as a risk factor for poorer sleep health in early childhood [14]. The authors did not include a measure of gendered racism, and thus did not acknowledge the possibility that for Black American women in particular, the simultaneous experience of racism and sexism may also be associated with poorer sleep health in their children. In the current study, we seek to replicate and extend the findings of Powell and colleagues by broadening the operational definition of pregnant Black American women’s exposure to racism to include measures of both racial/ethnic discrimination and gendered racial stress.

Powell and colleagues demonstrated that greater lifetime exposure to racial/ethnic discrimination, measured during early pregnancy, was associated with shorter 24 h sleep duration in women’s six-month-old infants [14]. While findings attenuated by age two, the authors noted several study limitations, including the use of a sample of pregnant women that reported high education and income levels. Experiences of discrimination vary based on these contextual factors [7], and studies that include participants from a range of socioeconomic backgrounds will better allow us to understand the effects of discrimination on health outcomes. Furthermore, Powell and colleagues relied solely on maternal reports of child sleep duration as their outcome measure of early childhood sleep health. Sleep duration is quite variable during the first years of life [15], and early childhood sleep health is a multidimensional construct [16]. We propose replicating Powell and colleagues’ work using a continuous measure of overall child sleep health that reflects multiple common clinical sleep concerns in toddlers, including sleep initiation, duration, night waking, and daytime drowsiness. Such an approach may allow us to better detect meaningful variation in healthy sleep in toddlers.

Empirical understanding of the potential mechanisms linking maternal racial/ethnic stress and children’s sleep health remains obscure. In this study, we will examine two prenatal factors that might explain this intergenerational risk process—maternal depressive symptoms and sleep quality during pregnancy. Black American women who report greater lifetime exposure to racial/ethnic discrimination report both greater depressive symptomatology [7,17] and poorer sleep quality during pregnancy [18]. Depressive symptoms and sleep difficulties occur within the broader social context and are elevated among pregnant women who identify as members of racial/ethnic minority groups and have experienced greater discrimination during their lifetimes [7,19]. Moreover, elevated maternal depressive symptomatology during pregnancy is associated with poorer sleep health in 18- and 30-month-old children [20]. On the basis of these previous findings, and in accordance with the Barker Hypothesis, we will examine maternal prenatal depressive symptoms and prenatal sleep quality as potential mediators of the association between pregnant Black American women’s lifetime exposure to racial/ethnic discrimination or gendered racial stress and sleep health in their two-year-old children.

Associations between greater maternal prenatal depressive symptoms and poorer offspring sleep have been documented in both animals and humans [21]. In preclinical studies, rats exposed to prenatal distress versus non-exposed controls experience greater sleep disturbances, three to four months post-birth [22,23]. Within the last 15 years, clinical research in human samples has extended animal work. Field and colleagues [24] sampled women (*n* = 253; 23% Black/African American) with and without a clinical diagnosis of depression during pregnancy and observed the sleep architecture of their newborn infants. Infants of mothers who carried a clinical diagnosis of depression spent less time in quiet sleep—a sleep stage that is similar to non-REM sleep in adults [25]. In a similar study design, researchers employed actigraphy to measure the sleep health of infants (*n* = 18; no participant race/ethnicity information provided). Infants born to mothers with clinical diagnoses of depression versus those without had longer sleep onset latency, lower sleep efficiency, and shorter sleep duration at two-weeks and six-months postpartum [26]. These findings are strengthened by complementary work in large, population-based cohorts, that studied sleep health later in childhood. Using maternal reports of offspring sleep, O’Connor and colleagues (*n* > 10,000; Avon Longitudinal Study of Parents and Children (ALSPAC) Cohort) demonstrated positive associations between higher levels of maternal prenatal depressive symptoms and greater sleep difficulties in 18- and 30-month-old children [20]. Associations persisted even after adjustment for women’s postnatal depressive symptoms. Similarly, Kim and colleagues (*n* = 5568; Growing Up in New Zealand cohort) found a twofold risk for more nighttime awakenings—as assessed by maternal report—among two-year-old children exposed to greater levels of maternal prenatal, but not postnatal, depressive symptoms [27]. Altogether, such findings indicate that greater prenatal depressive symptomatology increases the risk of poor sleep health in early childhood, even in the context of maternal postnatal depressive symptoms. However, and importantly, the literature in this area is lacking in minority health studies [28]. Indeed, women’s prenatal depressive symptomatology does not occur in a vacuum, but instead is subject to influence from contextual factors, such as racial/ethnic discrimination and gendered racial stress [4,7,17]. As such, it is important to examine these associations in the context of these factors for Black American women.

Finally, we will also explore prenatal sleep quality as a potential mediator in the association between women’s lifetime exposure to racial/ethnic discrimination or gendered racial stress and children’s sleep health at age two. Higher levels of lifetime exposure to racial/ethnic discrimination are associated with poorer sleep quality during pregnancy [18,19]. To our knowledge, few studies have examined associations between women’s prenatal sleep quality and their children’s sleep health, although preliminary evidence suggests positive associations between these constructs [29]. Given the co-occurrence between greater prenatal depressive symptomatology and poorer prenatal sleep quality [30], we will examine both of these constructs as mediators in a parallel mediator model [31]. Sleep quality and depressive symptomatology are typically correlated during pregnancy [30], and parallel mediator models assume correlation between variables. Moreover, parallel mediator models are robust—despite potential correlations between mediator variables, parallel mediator models prevent spurious indirect effects that may emerge simply because the mediator variables are correlated. In sum, we will examine both prenatal sleep quality and prenatal depressive symptoms as potential mediators of the association between women’s exposure to discrimination and children’s sleep health.

We have the following aims/hypotheses: first, (1) we predict that women who report greater lifetime exposure to racial/ethnic discrimination or gendered racial stress will report poorer sleep health in their two-year-old children, even after adjustment for women’s postnatal depressive symptoms and conceptually relevant covariates. Second, (2) we will examine maternal prenatal depressive symptoms and sleep quality as potential mediators of the association between lifetime exposure to racial/ethnic discrimination or gendered racial stress and child sleep health at age two. Findings from the current study will broaden our understanding of the ways in which expectant mothers’ distress is associated with the sleep health of their young children.

## 2. Materials and Methods

### 2.1. Participants

The current study constitutes a secondary data analysis of two prospective, longitudinal studies. Recruitment for both studies is ongoing and resulted in a sample of *N* = 205 women and children for current study analyses. All procedures were performed in accordance with the ethical standards of the Institutional Review Board and with the 1964 Helsinki declaration and its later amendments or comparable ethical standards. The first study (the Prenatal Study; R01NR014800) collected data from pregnant women at two timepoints during gestation. Healthy, pregnant women were recruited from prenatal clinics affiliated with two large hospitals—one private, one public—in a Southeastern city of the United States to participate in a study investigating the impacts of prenatal stress and environmental exposures on maternal and child health [32]. Inclusion criteria for enrollment in the Prenatal Study were as follows: Black/African American race/ethnicity (by self-report), singleton pregnancy, fluency in English, maternal age 18–40 years, and the absence of diagnoses of chronic health conditions (e.g., hypertension, diabetes) or chronic prescription medication use. When their children reached age two years, mothers whose pregnancies resulted in a live birth and whose infants were born without congenital disorders (e.g., cerebral palsy, spina bifida) were asked whether they would like to enroll in a follow-up study on children’s health outcomes (the Environmental Influences on Child Health Outcomes (ECHO) Study; 1UG3OD023318-01).

### 2.2. Procedure

During pregnancy, data were collected from participants twice: once between eight- and 14-weeks’ gestation (*M* = 11.16 weeks, *SD* = 2.25) and once between 24- and 30-weeks’ gestation (*M* = 26.59 weeks, *SD* = 2.73). At the first Prenatal Study visit (referred to as early pregnancy), women retrospectively reported on their lifetime exposure to racial/ethnic discrimination and their experiences of gendered racial stress and provided demographic and health information. At the second Prenatal Study visit (referred to as mid-pregnancy), women reported on their current prenatal sleep quality and depressive symptoms. When children were two years old, mothers and their children completed a two-hour study visit. At this time, mothers completed a questionnaire about their children’s sleep health and reported on their own depressive symptoms.

Inclusion criteria for the current study were (1) completion of at least one measure of discrimination in early pregnancy, (2) child gestational age at birth ≥32 weeks (which excludes children who would have been classified as very or extremely preterm and includes children who may have been classified as moderate or late preterm [33]), and (3) completion of the child sleep health measure at child age two.

### 2.3. Measures

#### 2.3.1. Lifetime Experiences of Racial/Ethnic Discrimination and Gendered Racial Stress

In early pregnancy, women retrospectively reported on their lifetime exposure to racial/ethnic discrimination using the *Experiences of Discrimination* measure (EOD) [34]. Women also reported on their experiences of gendered racial stress using the *Jackson, Hogue, Phillips Contextualized Stress* measure (JHP) [35].

EOD. The EOD asks participants to respond (yes/no) to the following question:

*“Have you ever experienced discrimination, been prevented from doing something, or been hassled or made to feel inferior in any of the following [nine] situations (i.e., at school, getting hired/getting a job, at work, getting housing, getting medical care, getting service at store/restaurant, getting credit/bank loans/mortgage, in public, with police/in courts) because of your race, ethnicity, or color?”*

Women’s responses on the EOD are limited to what they are willing to disclose, and do not necessarily represent the full range of discriminatory experiences. No items on the EOD reference sleep quality. Responses are summed to result in a total score (range = 0–9). This score is considered reliable and valid: it has good internal consistency, good test–retest reliability over a month timespan, and good convergent validity with other self-report measures of discrimination [34]. This measure of racial/ethnic discrimination has previously been used in work investigating pregnant Black American women’s exposure to discrimination as a predictor of psychological outcomes [7,14,36]. Internal consistency in the current study sample was good: Cronbach’s α = 0.81

JHP. The lived experience of Black American women is shaped by *both* racial/ethnic identity and gender identity [3,5]. As such, Black American women may be exposed to both racial/ethnic discrimination and gender-based discrimination, and these constructs are not wholly separable. The JHP is a 39-item self-report measure that assesses Black American women’s gendered racial stress. Participants indicate whether statements broadly describe their lived experience (0 = Strongly Agree, 1 = Agree, 2 = Unsure, 3 = Disagree, 4 = Strongly Disagree). Participants are not provided with a time anchor. Statements include, “*Everyone expects me to be strong for them*”, “*As an African American woman, I can withstand great pressure*”, “*Racism is a problem in my life*”, and “*I have to work harder than white women to earn recognition*”. The JHP is comprised of five subscales: burden, coping, racism, personal history, and work. Subscale scores are summed to result in a total summary score (range = 43–159). We tested current study hypotheses using summary scores from the JHP as a predictor variable, as we did not have a priori predictions about any particular subscale and wanted to tap the full construct of gendered racial stress. The JHP was developed via qualitative interviews with *n* ≥ 400 women in metropolitan Atlanta, and has good convergent validity with self-report measures of anger, depressive symptoms, and anxiety [35]. In the current study sample, internal consistency for the JHP total summary score was good: Cronbach’s α = 0.85.

#### 2.3.2. Prenatal Depressive Symptoms

In mid-pregnancy, women reported on their current depressive symptoms using summary scores from the *Edinburgh Postnatal Depression Scale* (EPDS) [37]. Participants decided whether 10 statements referencing low mood (e.g., “*I have been able to laugh and see the funny side of things.*”), suicidality (e.g., “*The thought of harming myself has occurred to me.*”), anhedonia (e.g., “*I have looked forward to things*.”), and stress (e.g., “*I have been anxious or worried for no good reason*.”) described them within the past week. Wording of response options varies by question, but is typically listed as: Yes, most of the time; Yes, quite often; Only occasionally; and No, not at all. One item on the EPDS directly references sleep and fatigue (“*I have been so unhappy that I have had difficulty sleeping.*”) and was removed from all analyses to avoid methodological overlap, resulting in a total of nine EPDS items. Scores on the EPDS range from 0 to 30; scores greater than or equal to 10 suggest clinically significant depressive symptoms in Black American perinatal women [38]. Continuous scores were used in all study analyses. In the current study sample, internal consistency for the 9-item scale was good: Cronbach’s α = 0.84. Internal consistency for the 10-item scale—without the sleep item removed—was similar: Cronbach’s α = 0.87.

#### 2.3.3. Prenatal Sleep Quality

Women also reported on their sleep quality in mid-pregnancy using the *Patient-Reported Outcomes Measurement Information System* (PROMIS) *Sleep Disturbance Short Form* [39]. This measure was developed in a sample of *N* = 2252 adult participants using Item Response Theory. Participants in the measure development sample (12.6% Black or African American) included those with and without clinically significant sleep quality concerns. The PROMIS is comprised of eight items that assess sleep quality within the past week (e.g., “My sleep was restless.”). No items directly reference mood symptoms or stress. Participants were asked to decide whether statements described their sleep quality (1 = not at all, 2 = a little bit, 3 = somewhat, 4 = quite a bit, 5 = very much). Responses are summed to compute a total continuous raw score (range = 8–40); higher scores indicate worse sleep quality. These continuous scores were used as our outcome measure in current study analyses. The PROMIS has high internal consistency and good convergent validity with other measures of sleep quality (i.e., *PROMIS Sleep Disturbance Full Form* [40], *Pittsburgh Sleep Quality Index* [41]; however, the PROMIS has greater precision in identifying adults with clinically significant sleep disturbances, and it has fewer items (i.e., eight versus 27 and 19, respectively), making it less burdensome [39]. This measure has previously been used in samples of perinatal women [42,43] although it has not, to our knowledge, been normed in this population. In the current study sample, internal consistency for the PROMIS was good: Cronbach’s α = 0.89.

#### 2.3.4. Maternal Depressive Symptoms at Child Age Two

When children were two years old, mothers repeated the EPDS [37] to assess their current depressive symptoms. In the current study sample, internal consistency for the 9-item scale was good: Cronbach’s α = 0.82. Internal consistency for the 10-item scale—without the sleep item removed—was similar: Cronbach’s α = 0.85.

#### 2.3.5. Child Sleep Health at Child Age Two

Mothers reported on their children’s sleep health at age two using the *Children’s Sleep Habits Questionnaire—Abbreviated* (CSHQ-A; [44]). The CSHQ-A contains 22 items and is a modified version of the 23-item *Children’s Sleep Habits Questionnaire—Short Form* (CSHQ—SF) [45]. Items on both measures assess behavioral aspects of child sleep health (e.g., difficulties falling and/or staying asleep, night wakefulness, sleep duration and regularity, daytime sleepiness) that are considered amenable to parent intervention. At child age two, mothers indicated how well (i.e., Always, Usually, Sometimes, Rarely, or Never) statements characterized their children’s sleep (e.g., “Child wakes up more than once during the night.”) during “the most recent typical week.” Higher scores on the CSHQ-A suggest poorer child sleep health, and serve as a screener for identifying children with clinical sleep difficulties [45]. In the current study sample, internal consistency was acceptable: Cronbach’s α = 0.70.

#### 2.3.6. Covariates

We selected covariates for inclusion a priori based on previous work investigating associations between women’s experiences of racial/ethnic discrimination [14] or prenatal depressive symptoms [20,27] and their young children’s sleep health. We included variables that have previously been associated with early childhood sleep health, our outcome variable, as covariates. O’Connor and colleagues demonstrated that younger maternal age and lower maternal education level were associated with greater sleep disturbances in children at ages 18 and 30 months [20]. In early pregnancy, women were asked to provide their age and their highest achieved education level and income. Education and income were averaged and standardized to form a composite socioeconomic status (SES) variable. SES is best captured by multiple factors rather than a single variable [46] and this methodological approach is consistent with previous research examining associations between discrimination and sleep health [47,48,49].

Further, O’Connor and colleagues hypothesized that greater prenatal alcohol and tobacco use would be associated with greater child sleep disturbances at ages 18 and 30 months. While they failed to find a significant association between these variables, recent work indicates significant associations between greater prenatal substance use and poorer early childhood sleep health [50]. Powell and colleagues also included prenatal tobacco use as a covariate in their models [14]. In the current study, at the early and mid-pregnancy study visits, women were asked to report whether they had used tobacco or alcohol at any point during pregnancy thus far. Women’s responses at both timepoints (0 = no, 1 = yes) were summed to result in a total count variable. Next, because healthy sleep differs by developmental stage [20,51], we adjusted for children’s age in months at the year two visit. In addition, we adjusted for the number of people in the home at child age two, consistent with O’Connor and colleagues’ measure of ‘crowding’ [20] and the idea that environmental context (e.g., greater noise) shapes sleep health [52]. Finally, to test for prenatal specific effects and to minimize reporter bias, we controlled for mothers’ depressive symptoms at child age two.

### 2.4. Data Analytic Plan

#### 2.4.1. Preliminary Analyses

All analyses were performed using IBM SPSS version 27.0 [53]. Statistical significance was set at a two-sided *p*-value of <0.05. A priori power analyses using G*Power [54] revealed that a sample size of *n* = 199 was needed to detect small to medium effects (Cohen’s *f* = 0.04; α = 0.05; *1* − β = 0.80; number of tested predictors = 1; total number of predictors = 7). We examined our data for missingness in compliance with the Journal Article Reporting Standards (JARS) for Research in Psychology [55]. To determine whether our data were Missing Completely at Random (MCAR), Missing at Random (MAR), or Missing Not at Random (MNAR) we dummy-coded variables to signify whether participants were missing data on study variables. Next, we conducted Independent Samples *t*-tests and Chi-Squared tests to determine whether missingness was associated with predictor, mediator, outcome, and/or sociodemographic variables. Finally, missing data were multiply imputed using the multiple imputation package in SPSS.

#### 2.4.2. Primary Analyses

We tested our study hypotheses using hierarchical linear regression models. To verify the assumptions of linear regression, we examined descriptive statistics and distribution fits for all included study variables. We plotted standardized residuals against standardized predicted values to inspect for linearity, homoscedasticity, and normality and examined our data for influential outliers. We used the PROCESS macro in SPSS [56] to test our mediation hypothesis. We performed two parallel mediation models, consistent with recommendations from Hayes and Rockwood [31]. We obtained 95% bias-corrected confidence intervals based on 10,000 bootstrap samples for all regression coefficients [57].

#### 2.4.3. Sensitivity Analyses

A portion of data collection occurred during COVID-19 pandemic. Given research suggesting poorer sleep quality [58] and depressive symptoms [59] among pregnant women and poorer sleep quality among children [60] during the early phases of the pandemic, we first examined whether women completed study visits during pregnancy after 1 March 2020. In the current study sample, all data collected during pregnancy was obtained prior to 1 March 2020. However, *n* = 42 mother–child dyads completed the child age two visit after 1 March 2020. We repeated all analyses with these participants removed. This resulted in a total of *N* = 163 mother–child dyads available for sensitivity analyses.

## 3. Results

### 3.1. Participants

There were limited missing data (4.53% of all values) in the current study sample. All women (*N* = 205) completed the racial/ethnic discrimination measure during early pregnancy, and all but one participant (*n* = 204) completed the gendered racial stress measure during early pregnancy. In mid-pregnancy, *n* = 179 women completed the sleep quality measure, and *n* = 180 women completed the measure of depressive symptoms. At child age two, all women (*N* = 205) completed the child sleep health measure. Participants who did not have available sleep quality data during mid-pregnancy participated in the mid-pregnancy visit earlier (*M* gestational weeks = 25.28, *SD* = 0.38) than participants who had available data on these constructs (*M* gestational weeks = 26.52, *SD* = 2.60, *t* = −4.26, *p* = 0.01). There were no other significant differences between these groups (all *p* > 0.05). We concluded that our data met Missing At Random (MAR) specifications [61]. Hereafter, all results reported in the text, Tables, and Figures are based on the multiply imputed data.

Table 1 displays descriptive statistics for variables in the current study sample. On average, mothers who participated in the current study were 25.1 years old *(SD* = 4.8 years) and for 37.1% of participants, this was their first pregnancy. The median highest level of education achieved was a high school diploma or General Educational Development (GED). Participants’ median income was less than 100–132% of the Federal Poverty Level. Table 2 displays bivariate correlations between variables in the current study sample. There was no significant association between women’s exposure to racial/ethnic discrimination and their child’s sleep health at age two; however, women’s experiences of gendered racial stress were positively associated with their child’s sleep health at age two. Greater gendered racial stress was prospectively associated with poorer child sleep health (*r* = 0.27, *p* < 0.001).

### 3.2. Main Effects of Women’s Exposure to Discrimination on Offspring Sleep Health

The results of simple linear regression analyses (Model 1 in Table 3 and Table 4) reflect the bivariate Pearson correlations displayed in Table 2. There was no significant association between women’s exposure to racial/ethnic discrimination and their child’s sleep health at age two (Δ*R*^2^ = <0.001, Δ*F* = 0.10, *p* = 0.76), whereas women’s experiences of gendered racial stress were positively associated with their child’s sleep health at age two (Δ*R*^2^ = 0.07, Δ*F* = 15.35, *p* < 0.001), such that greater gendered racial stress among women was associated with poorer sleep health in their children.

Next, we adjusted for conceptually relevant covariates and performed hierarchical multiple linear regression analyses (Models 2 and 3 in Table 3 and Table 4). In all cases, we observed an attenuation in effect sizes, but our pattern of findings remained the same. There was no association between women’s exposure to racial/ethnic discrimination and child sleep health at age two (Table 3). Next, we examined associations between women’s experiences of gendered racial stress and child sleep health at age two, with statistical adjustment for important health and environmental variables that may influence child sleep health (Model 2, Table 4; Δ*R*^2^ = 0.07, Δ*F* = 14.07, *p* < 0.001), and maternal depressive symptoms at child age two (Model 3, Table 4; Δ*R*^2^ = 0.05, Δ*F* = 11.15, *p* = 0.001). After adjustment for covariates, women’s experiences of gendered racial stress still explained a small, but statistically significant percentage of the variance in child sleep health at age two.

### 3.3. Examining Prenatal Sleep Quality and Prenatal Depressive Symptoms as Potential Mediators in the Association between Women’s Exposure to Discrimination and Child Sleep Health

**Figure 1:**
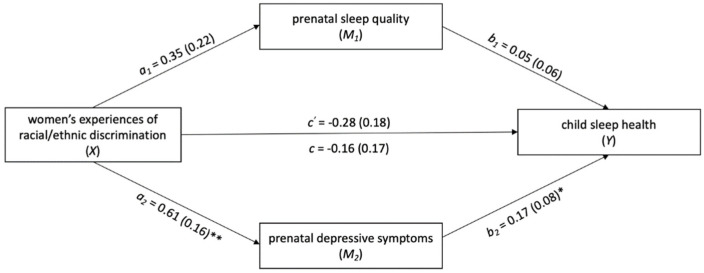
Examining prenatal sleep quality and depressive symptoms as parallel mediators of the association between women’s exposure to racial/ethnic discrimination and child sleep health at age two. Note. ** *p* < 0.01, * *p* < 0.05. Statistics displayed are unstandardized regression coefficients with standard errors in parentheses. The following covariates were included in the above mediation model: maternal age, SES, prenatal substance use, child age, number of people in the home at child age two, and maternal depressive symptoms at child age two. C′ represents the direct effect of X on Y, or the effect of X on Y while M_1_ and M_2_ are held constant. C represents the total effect of X on Y, which is the sum of the direct and indirect effects of X on Y. Indirect effects (A_1_*B_1_ and A_2_*B_2_) are described in-text.

**Figure 2:**
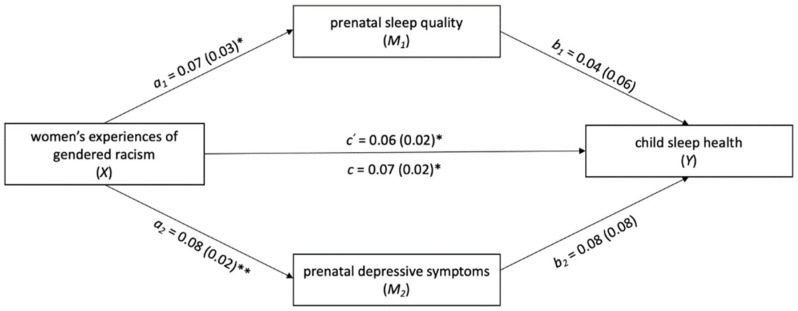
Examining prenatal sleep quality and depressive symptoms as parallel mediators of the association between women’s experience of gendered racial stress and child sleep health at age two. Note. ** *p* < 0.01, *p < 0.05. Statistics displayed are unstandardized regression coefficients with standard errors in parentheses. The following covariates were included in the above mediation model: maternal age, SES, prenatal substance use, child age, number of people in the home at child age two, and maternal depressive symptoms at child age two. C′ represents the direct effect of X on Y, or the effect of X on Y while M_1_ and M_2_ are held constant. C represents the total effect of X on Y, which is the sum of the direct and indirect effects of X on Y. Indirect effects (A_1_*B_1_ and A_2_*B_2_) are described in-text.

PROCESS analyses partially supported the hypothesized mediation model (Figure 1 and Figure 2). There was evidence of a small, but significant indirect effect of prenatal depressive symptoms on the association between women’s exposure to racial/ethnic discrimination and child sleep health at age two (*B* = 0.10, *SE* = 0.06; 95% *CI B* [0.001–0.23]). The indirect effect accounted for 65.0% of the association between women’s exposure to racial/ethnic discrimination and child sleep health. Prenatal sleep quality did not mediate this association (*B* = 0.02, *SE* = 0.03; 95% *CI B* [−0.03–0.08]).

In contrast, there was no evidence of a significant indirect effect of either prenatal sleep quality (*B* = 0.002, *SE* = 0.004; 95% *CI B* [−0.01–0.01]) or prenatal depressive symptoms (*B* = 0.01, *SE* = 0.01; 95% *CI B* [−0.01–0.02]) on the association between women’s experience of gendered racial stress and child sleep health at age two.

### 3.4. Sensitivity Analyses

Results of sensitivity analyses, in which we limited analyses to data points collected prior to the COVID-19 pandemic, indicated the same pattern of findings for both study hypotheses. This suggests that the inclusion of data collected during the early COVID-19 pandemic did not influence our findings (data not shown).

## 4. Discussion

The results of the current study suggest that pregnant Black American women’s own experiences of gendered racism are associated with poor sleep health in their two-year-old children. We also found preliminary evidence suggesting that the women’s exposure to racial/ethnic discrimination indirectly influences children’s sleep health via increases in women’s depressive symptoms during pregnancy. Our findings are consistent with previous work demonstrating that pregnant women who report higher levels of depressive symptoms report more sleep difficulties in their toddlers, even after adjustment for postnatal depressive symptoms [20]. In addition, pregnant women who report greater lifetime exposure to racial/ethnic discrimination report shorter sleep duration in their infants, even after adjustment for postnatal depressive symptoms [14]. Chronic exposure to interpersonal discrimination is associated with poorer health outcomes among adults [1] and children [62]. In the current study, we expanded the definition of exposure to discrimination by applying an intergenerational transmission of racism framework [63] to investigate how women’s own experiences being discriminated against may have bearing on the health of her young child. In doing so, we demonstrated that women’s interpersonal exposure to racism and gendered racism is associated with poor sleep health in their two-year-old children.

Our findings varied by type of interpersonal exposure to discrimination. We did not find evidence of a direct association between women’s lifetime exposure to racial/ethnic discrimination and two-year-old children’s sleep health. However, we found evidence for a small, but significant indirect effect between these constructs. As hypothesized, we demonstrated that women’s prenatal depressive symptomatology mediated the positive association between greater lifetime exposure to racial/ethnic discrimination and poor sleep health in women’s two-year-old children. Prenatal depressive symptomatology occurs within the broader sociocultural context and is elevated among pregnant women who have experienced higher levels of racial/ethnic discrimination throughout their lifetimes [7,17]. As mentioned previously, findings from pre-clinical and clinical study samples indicate that higher levels of prenatal stress and depressive symptomatology, respectively, are associated poorer sleep health in offspring [21]. Elevated levels of stress experienced by pregnant women may be transmitted to their offspring through biological mechanisms that occur *in utero.* The prenatal specificity of these effects is consistent with the Barker Hypothesis [8]. One potential biological mechanism of transmission may be as follows: increased stress and/or elevated depressive symptoms during a woman’s pregnancy may alter gene expression that dictates how permeable the placenta is to glucocorticoids produced by the mother [21]. With the placenta more susceptible to glucocorticoid exposure, cortisol—rather than the benign cortisone—crosses the placental barrier, triggering alterations in fetal neural functioning and shaping aspects of infant and early childhood health [64]. Supporting this hypothesis, recent findings from a study of fetal neural development indicate links between elevated prenatal maternal stress and sleep difficulties in women’s three-year-old children. Elevated prenatal maternal stress was also associated with decreased fetal neural connectivity in the cerebellum, which the authors originally proposed as a mechanism [65]. The authors did not find evidence of an indirect effect, but they suggested that different neural connectivity pathways in the fetal brain may underly links between maternal prenatal psychological distress and children’s sleep in the first few years of life.

To our knowledge, the current study is the first to examine maternal prenatal depressive symptomatology as a mediator in the association between women’s exposure to racial/ethnic discrimination and their young children’s sleep health. We sought to first establish this relationship before examining potential biological mechanisms of transmission that may underly these associations. We encourage future researchers to expand upon findings from the current study by examining biological mediators that may serve as physiological markers of pregnant women’s stress and/or mood symptoms and may explain the association between women’s exposure to racial/ethnic discrimination and their two-year-old children’s sleep health.

Our second mediation hypothesis—that women’s prenatal sleep quality would also mediate associations between racial/ethnic discrimination and children’s sleep health—was not supported. To our knowledge, few studies have examined links between pregnant women’s sleep and their children’s sleep [29]. Based on findings from previous research that greater interpersonal exposure to racial/ethnic discrimination is associated with poorer sleep quality during a woman’s pregnancy [18], and known associations between prenatal sleep quality and depressive symptoms [30], we hypothesized that women’s mood and/or sleep may mediate associations between women’s lifetime exposure to racism and their children’s sleep health. In the current study, there was a moderate correlation between pregnant women’s depressive symptoms and their sleep quality. Our use of a parallel mediator model accounted for the correlation between these constructs, and prenatal depressive symptomatology was ultimately found to be a stronger mediator variable. However, given positive associations between women’s exposure to discrimination and poorer sleep quality in pregnancy, future researchers may also consider examining objective measures of women’s sleep health during pregnancy as potential mediator variables. Our measure of prenatal sleep quality has not been validated among pregnant women despite being used in several studies of perinatal health [43,66]. The use of objective prenatal sleep data may be needed to more fully assess this potential mediator. In addition, prenatal women’s sleep quality cannot be fully explained by contextual life stressors (i.e., discrimination). During pregnancy, sleep quality declines among all women due to typical changes in body morphology and physiology that occur across gestation [67]. Future studies examining associations between women’s exposure to discrimination and their sleep quality during pregnancy may consider including additional measures of women’s health as potential confounds, to better isolate the hypothesized associations. In sum, in the current study, while women’s exposure to racial/ethnic discrimination was associated with poorer sleep quality during pregnancy, prenatal sleep quality did not mediate associations between women’s experiences of racism and their two-year-old children’s sleep health.

Consistent with recommendations that researchers apply an intersectionality framework to better understand associations between discrimination and sleep health [68], we additionally examined associations between Black American women’s exposure to gendered racism and their two-year-old children’s sleep health. There was a significant, direct effect between these constructs, such that higher levels of reported gendered racial stress were associated with poorer sleep health in women’s two-year-old children, even after adjustment for important covariates, including women’s depressive symptoms when children were two years old. We did not find evidence that either women’s prenatal sleep quality or depressive symptomatology mediated this association. This suggests that other factors may explain the association between these constructs.

Our proposed mechanisms of transmission were specific to the prenatal period, but it is entirely possible that postnatal factors may mediate the association between women’s exposure to gendered racism and their two-year-old children’s sleep health. In the only known study to examine similar associations, Powell and colleagues hypothesized that greater exposure to racial/ethnic discrimination may be associated with a decreased ability to provide warm and responsive parenting [14]. Parenting behaviors may mediate the association between women’s exposure to racism, or in the case of the current study, gendered racism, and poorer sleep health in toddler age children. For instance, women who report elevated psychological distress experience poorer sleep themselves, which is in turn associated with lower levels of warm, responsive parenting [69]. Lower levels of warm and responsive maternal parenting behaviors at bedtime are associated with poorer sleep health in infants (e.g., shorter sleep duration, more night awakenings; [70]), consistent with the idea that young children may benefit from parents’ emotional availability to learn how to self-soothe and sleep well [71]. The ability to provide consistent warm and responsive parenting behaviors may be compromised among women who experience significant psychological burden and demand, such as women exposed to higher levels of gendered racism. In keeping with the Barker Hypothesis [8], in the current study, we conceptualized our mediator variables as occurring in the context of pregnancy and transmitting effects to children’s sleep health via intrauterine physiological processes. As such, we did not examine potential postnatal exposures or mechanisms of transmission. While two-year-old children’s sleep health may be shaped by prenatal processes [20], it is also likely that more proximal postnatal environmental variables such as parenting behaviors have bearing on young children’s sleep. We recommend that future researchers examine parenting behaviors as a potential mediator in the association between women’s experiences of gendered racism and their two-year-old children’s sleep health.

### 4.1. Limitations

The current study is not without limitations. First, we assessed women’s exposure to racial/ethnic discrimination and gendered racism using measures that consider interpersonal exposures. We did not specifically assess for women’s exposure to institutional racism and/or gendered racism, despite evidence that societal and structural factors are associated with poorer prenatal health in women [72] and poorer sleep health in children [71,72]. For instance, infants living in neighborhoods that have a high percentage of residents whose highest education level is a high school diploma, whose incomes are below the national poverty line, and receive federal assistance have poorer sleep quality [73,74,75]. In the current study, we were primarily interested in Black American women’s interpersonal exposure to racial/ethnic discrimination as we aimed to replicate the only—to our knowledge—previous study that examined associations between this construct and children’s sleep health [14]. Women’s reports of interpersonal exposure to racism are predictive of greater depressive symptomatology during pregnancy [7,17] and highlight women’s understanding perceptions based on race, ethnicity, or skin color. Similarly, our measure of women’s gendered racial stress is by nature interpersonal [35]. We did control for contextual factors such as mothers’ SES (i.e., income, education level) and the number of people living in the home when children were two years old, given associations between each of these constructs and children’s sleep health. However, our knowledge concerning the intergenerational impacts of discrimination on children’s sleep health would be further strengthened by incorporating indices of institutional discrimination, such as neighborhood census tracts.

A second limitation is that all measures were self-report questionnaires completed by participants. Measuring women’s experiences of racism, gendered racism, prenatal depressive symptomatology, prenatal sleep quality, and toddler sleep health via maternal self-report may have resulted in shared method variance. In other words, women who have experienced greater stress or are more depressed may answer questions about themselves or their children’s behavior with a negative cognitive bias [76]. To account for the possibility that women predisposed to more negative emotionality may have reported worse sleep in their two-year-old children regardless of children’s actual sleep health, we adjusted for women’s depressive symptoms when children were two years old in all study analyses. This allowed us to isolate the effects of women’s exposure to racial/ethnic discrimination or gendered racism—as measured during pregnancy—even in the context of women’s mood state at the time of the age two study visit. Relatedly, the current study would have been further strengthened had we utilized objective measures of children’s sleep health such as actigraphy. The current study constituted a secondary data analysis of a larger study that did not include additional measures of children’s sleep health. While maternal report of children’s sleep health is informative, it may retain an aspect of bias and does not capture all aspects of children’s sleep [16], particularly if children are sleeping in a different room than their mother, which may be the case for two-year-old children in the current study sample. We recommend that future studies examining associations between women’s exposure to racism or gendered racism and their children’s sleep health include both maternal report of child sleep as well as objective sleep data.

### 4.2. Strengths

Despite these limitations, the current study has several strengths. First, our measure of racial/ethnic discrimination has previously been used in a sample of pregnant Black American women to examine associations between discrimination and prenatal depressive symptoms [7]. In addition, this measure was used as a predictor variable in the only known—to our knowledge—study to previously investigate associations between women’s exposure to discrimination and their young children’s sleep health [14]. Therefore, our use of the Experiences of Discrimination measure [34] allows us to situate the current study within the extant literature on pregnant women’s lifetime exposure to racism and maternal child health outcomes. Next, we additionally explored all study hypotheses using the Jackson, Hogue, Phillips Contextualized Stress measure. This measure was borne out of intersectionality theory [2,3], and was developed to specifically understand the ways in which racism and sexism overlap and simultaneously shape Black American women’s health and wellbeing. The use of this measure of gendered racial stress strengthens the current study. It allows us to consider how discriminatory stress specific to Black American women’s dual, interlocking racial/ethnic and gender identities may confer risk for their health and the health of their two-year-old children.

Additionally, our assessments of racial/ethnic discrimination and gendered racism are not time-bound and instead assess women’s exposure to discrimination across the life course, until the time of assessment during pregnancy. We were unable to obtain data on when these exposures occurred in relation to women’s pregnancies, and thus cannot state whether there are sensitive periods during which women may be particularly vulnerable to discrimination. However, this approach is consistent with recommendations from Gee and colleagues [77], who assert that exposure to discrimination is cumulative. That is, earlier exposures shape individuals’ vulnerability to subsequent exposures. It is the collective sum of these exposures that is associated with health outcomes, rather than any one exposure in isolation. Finally, we took a novel approach to understand whether women’s experiences of discrimination may be associated with their two-year-old children’s sleep health, at least partly via women’s prenatal depressive symptoms. While previous research has thoroughly examined associations between pregnant women’s mood symptoms and their infant and toddlers’ sleep health [20], to our knowledge, only one study to date has applied an intergenerational transmission of racism approach to examine associations between pregnant women’s exposure to discrimination and their children’s sleep health [14]. In the current study, we united two previously siloed studies to examine preconception and prenatal influences on two-year-old children’s sleep health. We hope that future research will further expand upon our findings.

## 5. Conclusions

The current study adds to the growing literature on the intergenerational transmission of the effects of racism on pregnant Black American women to their young children [63] Black American women’s exposure to gendered racism is associated with poorer sleep health in their two-year-old children, and that Black American women’s exposure to racial/ethnic discrimination is associated with poorer sleep health in their children, at least partly via women’s depressive symptoms in mid-pregnancy. These findings highlight the importance of deliberately attending to the experiences of Black American women who present for prenatal care. Black American women are particularly vulnerable to both racism and gendered racism and these stress exposures carry risk for their own physical and mental health during pregnancy, as well as their children’s sleep health, a transdiagnostic marker of overall pediatric health. Prenatal care providers should sensitively screen for and inquire about women’s exposure to discrimination with the understanding that women who report higher levels of exposure may derive particular benefit from culturally sensitive interventions that target psychological outcomes and overall wellbeing. For instance, women who report higher levels of exposure to discrimination may be good candidates for CenteringPregnancy, a group based prenatal intervention that has been linked to better maternal child health outcomes in Black American women and their infants [78]. More broadly, larger structural factors perpetuate discrimination and put Black American women at risk for poorer health outcomes [12]. Both individual and structural level changes are needed to truly mitigate consequences. Eliminating racism and gendered racism will benefit the health of women and have downstream effects on the health of their children, thereby breaking harmful intergenerational cycles of risk.

## Figures and Tables

**Table 1:** Descriptive statistics.

Variable	Imputed Data (*N* = 205)
	*M* (*SD*) or *n* (%)
Maternal Age (years)	25.11 (4.75)
Primiparous Women	76 (37.1%)
In a Relationship and Cohabitating	98 (47.8%)
Education	-
8th Grade or Less	1 (0.5%)
Some High School	36 (17.6%)
Graduated High School or GED	81 (39.5%)
Some College or Technical School	52 (25.4%)
Graduated College	26 (12.7%)
Some Graduate Work or Degree	9 (4.4%)
Income	-
<100% of the Federal Poverty Level	97 (47.3%)
100–132% of the Federal Poverty Level	37 (18.0%)
133–149% of the Federal Poverty Level	11 (5.4%)
150–199% of the Federal Poverty Level	24 (11.7%)
200–299% of the Federal Poverty Level	19 (9.3%)
300–399% of the Federal Poverty Level	6 (2.9%)
≥400% of the Federal Poverty Level	11 (5.4%)
Prenatal Substance Use	-
Tobacco	30 (14.6%)
Alcohol	14 (6.8%)
Racial/Ethnic Discrimination (EOD) ^a^	2.09 (2.28)
Gendered Racial Stress (JHP) ^b^	96.45 (19.57)
Maternal Depressive Symptoms (EPDS) ^c^, mid-pregnancy	7.54 (5.64)
Maternal Sleep Quality (PROMIS) ^d^, mid-pregnancy	21.78 (7.16)
Gestational Weeks at Birth	38.69 (1.52)
Child Sex	102 female (49.8%)
Child Age (months)	25.84 (2.76)
Child Sleep Health (CSHQ), age two ^e^	36.34 (5.43)
Maternal Depressive Symptoms (EPDS), child age two	5.58 (4.85)
Number of People in the Home, child age two	4.34 (1.60)

*Note*. ^a^ The EOD was completed in early pregnancy; scores range from 0 to 9. ^b^ The JHP was completed in early pregnancy; scores range from 43 to 159. ^c^ The EPDS was completed in mid-pregnancy and again at child age two. EPDS scores range from 0 to 30; in racial/ethnic minority women, scores greater than or equal to 10 suggest clinically significant depressive symptomatology. EPDS means reported here include the sleep item, but when using EPDS summary scores in all study analyses, the sleep item was removed. Bivariate correlations between the 9- and 10-item EPDS scores were high (*r* = 0.99, *p* < 0.001 at both study timepoints). ^d^ PROMIS scores range from 8 to 40; scores greater than or equal to 25 suggest mild sleep disturbance. ^e^ CSHQ scores range from 26 to 58; higher scores indicate poorer sleep health.

**Table 2:** Correlations between study variables (*N* = 205 for all cells) ^a^.

	1	2	3	4	5	6	7	8	9	10	11
(1) Maternal Age	-										
(2) SES	0.42 **	-									
(3) Prenatal Tobacco Use	0.01	−0.18 *	-								
(4) Prenatal Alcohol Use	0.15 *	0.06	0.33 **	-							
(5) Racial/Ethnic Discrimination	0.11	0.16 *	0.04	0.09	-						
(6) Gendered Racial Stress	0.12	−0.06	0.05	0.15 *	0.26 **	-					
(7) Sleep Quality, mid-pregnancy	0.13	0.09	0.07	0.03	0.18 **	0.23 **	-				
(8) Depressive Symptoms, mid-pregnancy	0.02	−0.02	0.10	0.07	0.31 **	0.36 **	0.44 **	-			
(9) Child Sleep Health	0.11	−0.03	0.07	0.04	−0.02	0.27 **	0.15 **	0.20 **	-		
(10) Child Age (in months, age two)	−0.06	0.05	−0.09	−0.13	0.01	0.03	0.19 **	0.12	−0.07	-	
(11) Number of People in the Home, child age two	−0.02	−0.25 **	−0.01	−0.02	0.03	−0.09	0.01	−0.06	0.03	0.02	-
(12) Maternal Depressive Symptoms, child age two	0.001	−0.03	0.06	−0.10	0.20 **	0.20 **	0.26 **	0.40 **	0.15 *	0.13	0.08

*Note*. ** *p* < 0.01, * *p* < 0.05. ^a^ Prenatal Tobacco Use and Prenatal Alcohol Use are ordinal variables; all other study variables are continuous. Correlations performed between only continuous study variables are bivariate Pearson correlations; correlations between ordinal and continuous study variables are point-biserial correlations; the correlation between the two ordinal study variables is a Kendall Tau-beta correlation.

**Table 3:** Child sleep health at age two regressed on women’s exposure to racial/ethnic discrimination (*N* = 205).

	*B (SE)*	*95% CI (B)*	*β*	*t*	*p*
Model 1. Unadjusted Effects of Racial/ Ethnic Discrimination	−0.05 (0.17)	[−0.38–0.28]	−0.02	−0.31	0.76
Model 2. Effects of Racial/Ethnic Discrimination + adjustment for maternal age, SES, prenatal substance use, child age, number of people in the home at child age two	−0.07 (0.17)	[−0.41–0.27]	−0.03	−0.41	0.68
Model 3. Effects of Racial/Ethnic Discrimination + adjustment for maternal age, SES, prenatal substance use, child age, number of people in the home at child age two, maternal depressive symptoms at child age two	−0.16 (0.17)	[−0.50–0.19]	−0.07	−0.90	0.37

**Table 4:** Child sleep health at age two regressed on women’s experience of gendered racial stress (*N* = 205).

	*B (SE)*	*95% CI (B)*	*β*	*t*	*p*
Model 1. Unadjusted Effects of Gendered Racial Stress	0.07 (0.02)	[0.04–0.11]	0.27	3.92	<0.001
Model 2. Effects of Gendered Racial Stress + adjustment for maternal age, SES, prenatal substance use, child age, number of people in the home at child age two	0.07 (0.02)	[0.04–0.11]	0.26	3.75	<0.001
Model 3. Effects of Gendered Racial Stress + adjustment for maternal age, SES, prenatal substance use, child age, number of people in the home at child age two, maternal depressive symptoms at child age two	0.07 (0.02)	[0.03–0.11]	0.24	3.34	0.001

## Data Availability

Participants of the current study did not consent for their data to be shared with journal outlets, so supporting data is not available.
